# Retraction Note: Analysis of protein-protein interaction network and functional modules on primary osteoporosis

**DOI:** 10.1186/s40001-015-0128-2

**Published:** 2015-03-26

**Authors:** Gai-Li Li, Xian-Hua Xu, Bing-Ang Wang, Yi-Min Yao, Yang Qin, Shu-Rong Bai, Jian Rong, Tao Deng, Yong-He Hu

**Affiliations:** Department of Geriatrics, Chengdu Military General Hospital, No. 270, Rongdu Avenue, Jinniu District, 610083 Chengdu, China; Department of Orthopedics, 452 Hospital of the People’s Liberation Army, Shunjiang Road, 610021 Chengdu, China

## Retraction

The Publisher and Editor regretfully retract this article [1] because the peer-review process was inappropriately influenced and compromised. As a result, the scientific integrity of the article cannot be guaranteed. A systematic and detailed investigation suggests that a third party was involved in supplying fabricated details of potential peer reviewers for a large number of manuscripts submitted to different journals. In accordance with recommendations from COPE we have retracted all affected published articles, including this one. It was not possible to determine beyond doubt that the authors of this particular article were aware of any third party attempts to manipulate peer review of their manuscript.
